# Primary Intracranial Solitary Fibrous Tumor With Metachronous Pulmonary and Bone Metastasis: A Case Report

**DOI:** 10.7759/cureus.32607

**Published:** 2022-12-16

**Authors:** Haila Alabssi, Maram Alismail, Mahmoud S Taha, Marwah M Abdulkader, Nedal Bukhari

**Affiliations:** 1 Department of Internal Medicine, Imam Abdulrahman Bin Faisal University, Dammam, SAU; 2 Department of Neurosurgery, King Fahad Specialist Hospital, Dammam, SAU; 3 Department of Pathology and Laboratory Medicine, King Fahad Specialist Hospital, Dammam, SAU; 4 Department of Medical Oncology, King Fahad Specialist Hospital, Dammam, SAU

**Keywords:** case report, tumor, metastasis, intracranial, solitary fibrous tumor

## Abstract

An intracranial solitary fibrous tumor (SFT) is a rare and aggressive tumor with a high propensity for locoregional recurrence and distant metastasis. The formerly used collective term for this tumor, "solitary fibrous tumor/hemangiopericytoma", has recently fallen out of use and is now commonly replaced with the term "solitary fibrous tumor". We describe a rare case of intracranial SFT with simultaneous metastasis to the spine, the right humerus, and the lungs four years after resection and radiotherapy of the primary tumor.

## Introduction

In the 2021 edition of the WHO Classification of Tumors of the Central Nervous System (CNS), the term "hemangiopericytoma" has been retired, and the tumor is now referred to as "solitary fibrous tumor" (as opposed to the hybrid term "solitary fibrous tumor/hemangiopericytoma" used in the 2016 CNS classification) [1]. This terminology is consistent with the nomenclature for soft tissue pathology and strongly emphasizes the biological similarities within tumor types [1]. Intracranial SFT was first reported in 1996 and is still extremely rare, constituting 2.5% of meningeal neoplasms and less than 1% of all intracranial tumors [2,3]. The tumor shares analogous radiological and clinical features with meningiomas making histologic confirmation the only definitive way to distinguish the two entities [4]. Meanwhile, SFT is known for its relentless tendency for local recurrence and extracranial metastases [2]. This report describes a rare case of bilateral lung and bone metastasis from an intracranial solitary fibrous tumor discovered four years after resecting the primary tumor.

## Case presentation

**Figure 1:**
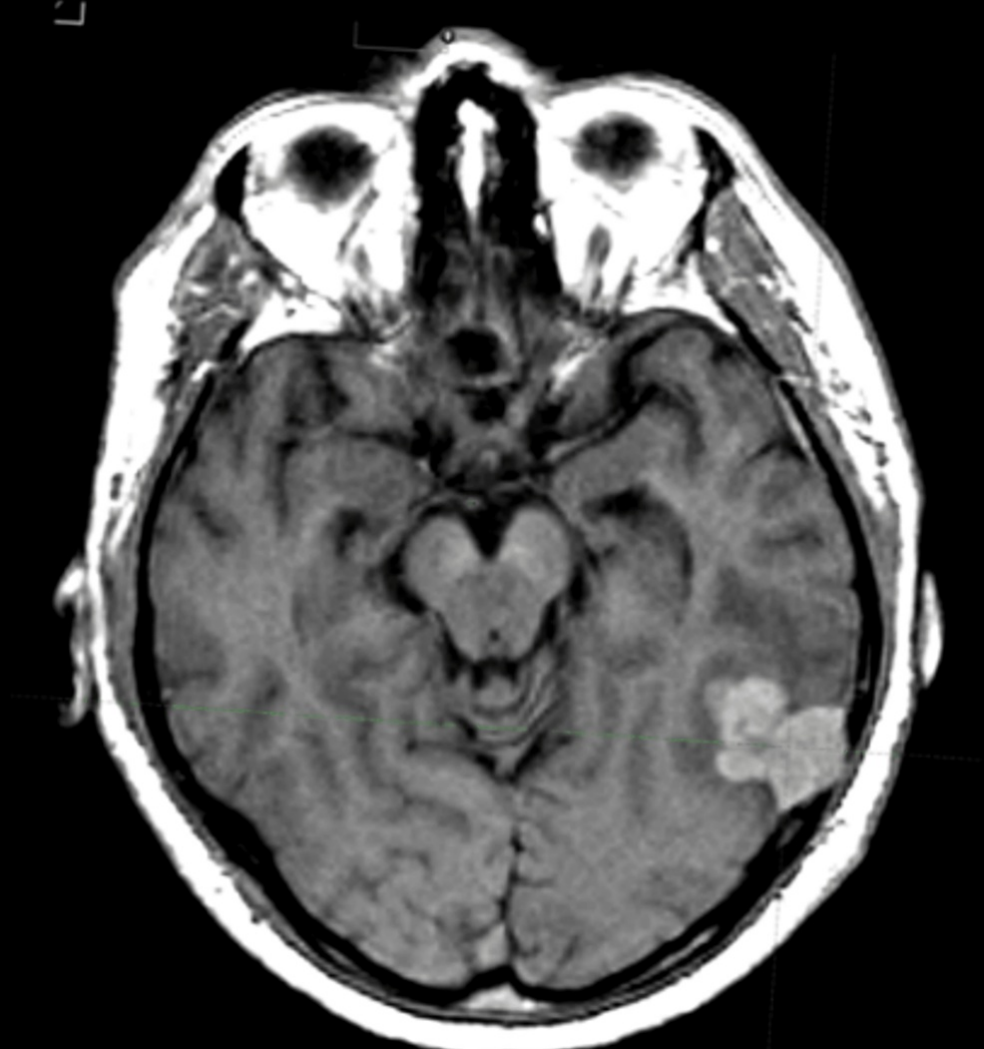
Preoperative magnetic resonance examination performed on the axial plane showing an extra-axial mass measuring 2x3cm localized on the left parietooccipital area.

A 65-year-old man presented to the emergency department of another institution with an episode of tonic-clonic seizure and loss of consciousness. Before his presentation, he complained of mild chronic headaches for the past two years. Diagnostic workup, including magnetic resonance imaging (MRI), and computed tomography (CT), revealed a 2x3cm left parietooccipital lesion invading the skull bone (Figure 1). Thereafter, the patient underwent a left parietooccipital craniotomy and total tumor resection. 

**Figure 2:**
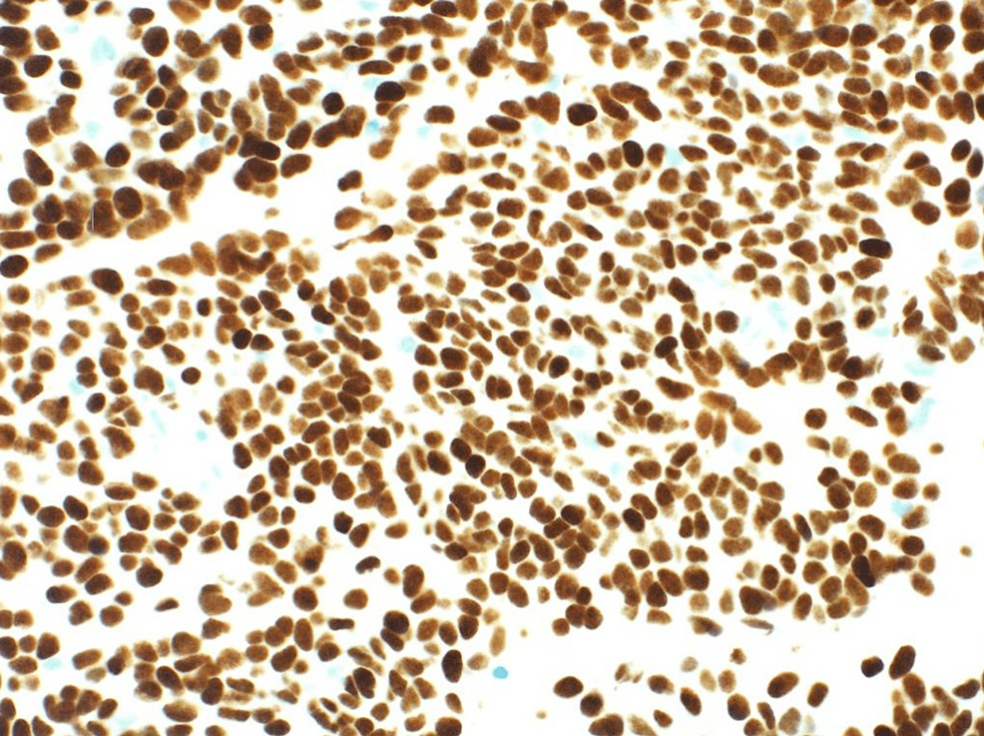
Immunostaining with STAT6 showing diffuse strong positive staining of the neoplastic cells.

**Figure 3:**
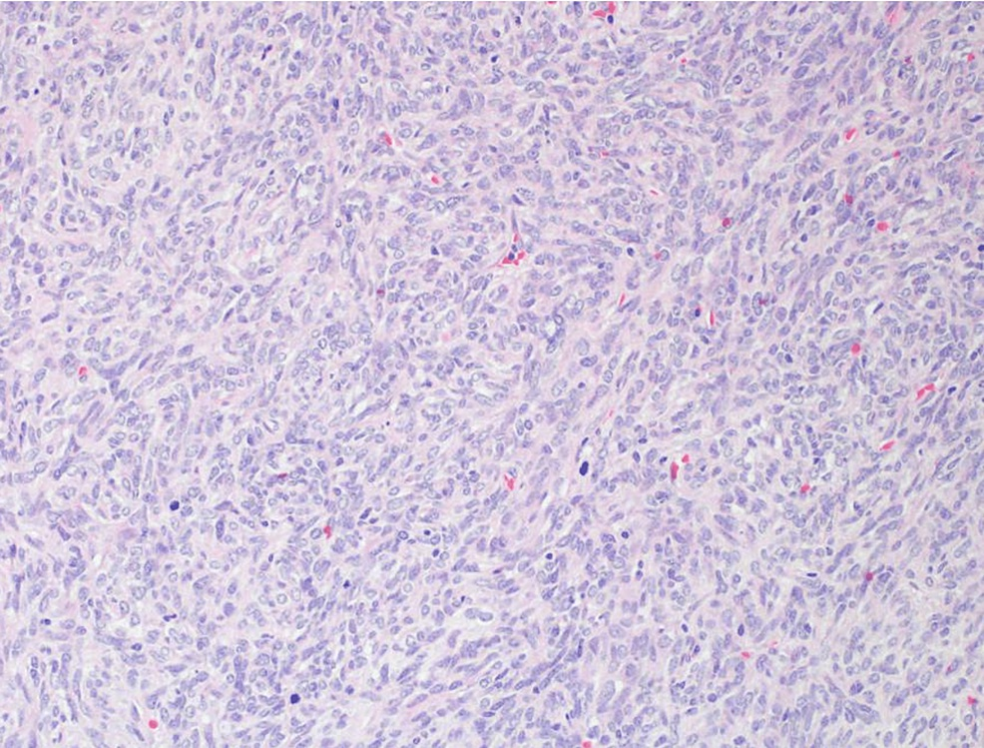
Hematoxylin and eosin showing short haphazard fascicles of spindle cells with many mitotic figures.

Histological examination revealed an extra-axial well-circumscribed hypercellular spindle cell neoplasm, composed of ovoid to fusiform cells with indistinct cell borders arranged in short haphazard fascicles in a collagenous stroma. The vessels displayed a prominent hemangiopericytomatous-like pattern with its characteristic dilated branched and staghorn-like morphology. Foci of nuclear pleomorphism and small areas of necrosis were present, and the mitotic count was high (18 mitoses/10 high-power field). Immunohistochemical staining showed CD34, BCL2, CD99, and STAT6 immunoreactivity. Epithelial membrane antigen (EMA), pancytokeratin, glial fibrillary acidic protein (GFAP), and S100 were all negative. The final pathological diagnosis was solitary fibrous tumor, CNS WHO grade 3 (Figures 2-3). 

After surgery, the patient was referred to our oncology services for further treatment and follow-up. Postoperative radiological imaging, including CT, MRI, and positron emission tomography (PET) scan, confirmed the absence of residual disease, recurrence, or distant metastasis. Adjuvant radiotherapy with a total dose of 60 Gy was administered to complete the treatment in addition to MRI surveillance every six to 12 months. 

**Figure 4:**
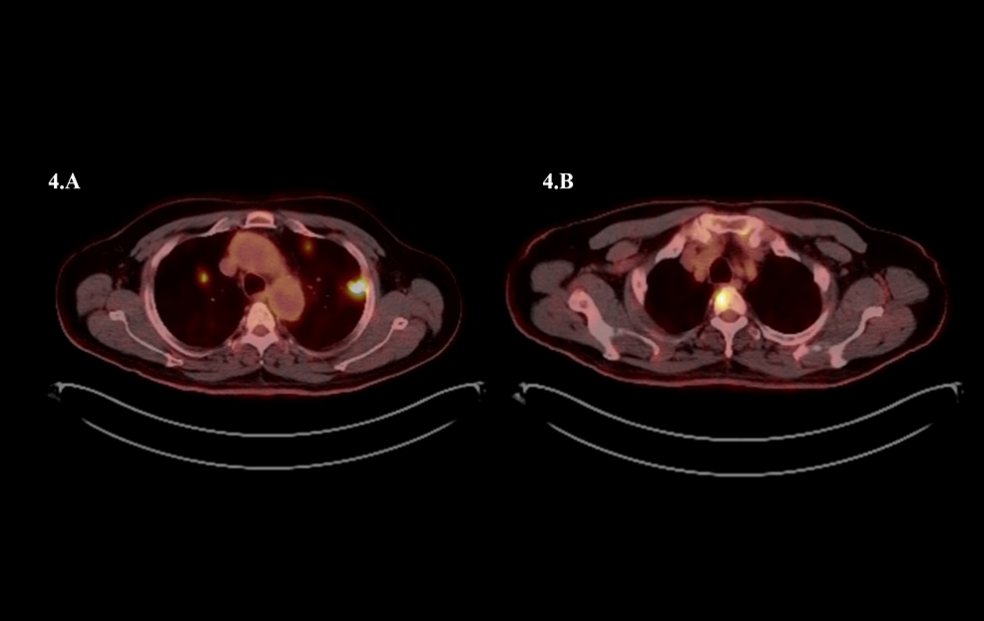
Positron emission tomography (PET) scan showing multiple F-fluorodeoxyglucose (FDG) avid pulmonary nodules (A) and bony lesions in the second thoracic vertebrae (B)

**Figure 5:**
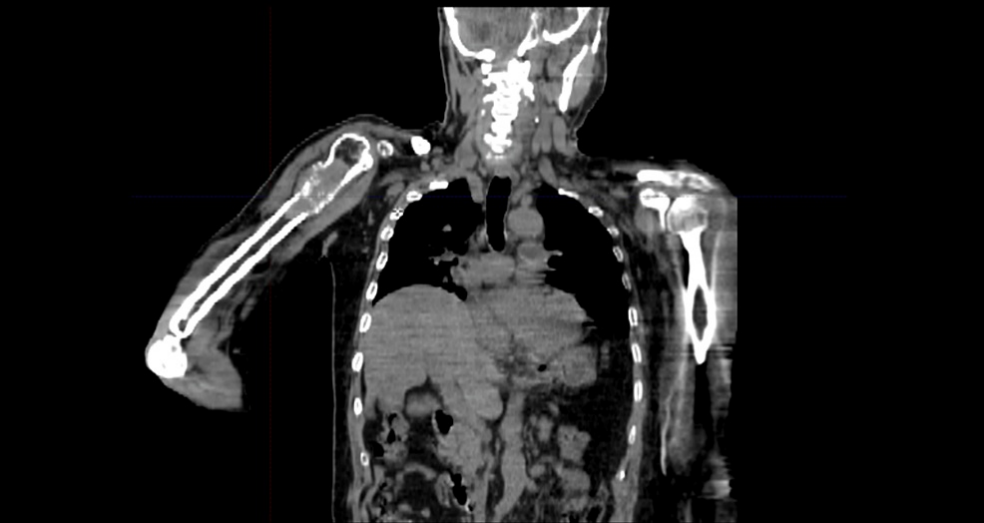
Computed tomography (CT) showing expansile lesion in the upper third of the right humeral bone.

Four years later, the patient sought medical advice for right elbow pain which prompted a visit to his local hospital. An X-ray of the elbow showed a soft tissue mass, thereafter he was referred back to his neurosurgeon. CT of the abdomen, chest, and pelvis (CAP) was significant for multiple bilateral pulmonary nodules. We then performed a PET scan, showing multiple F-fluorodeoxyglucose (FDG) avid pulmonary nodules and bony lesions in the second thoracic vertebrae (Figure 4) and right humerus (Figure 5).

The lung core biopsy showed lung parenchyma infiltrated by a metastatic spindle to an epithelioid high-grade tumor, the neoplastic cells were mildly pleomorphic in a collagenous background. The vessels were branched and the overall histological pattern was similar to that seen previously in the meningeal tumor. The cells were immunoreactive with STAT 6, CD34, BCL2, and CD99. Thyroid transcription factor 1 (TTF1), synaptophysin, CD56, and pancytokeratin were all negative. Later one NAB2-STAT6 gene fusion was detected in next-generation sequencing to confirm metastasis of solitary fibrous tumor.

The case was treated as a stage IV soft tissue sarcoma. Consequently, the patient received six cycles of doxorubicin/ifosfamide-based chemotherapy. In addition, he received palliative radiotherapy (8 Gy in fractions) for his right elbow. Upon follow-up in 2022, the patient had a five-month break from medications as the disease and the brain MRI were stable. However, PET-CT showed disease progression in the lungs, so he was started on dose-reduced pazopanib at 600 mg daily. Disease progression was noted to his lung metastasis after eight months of pazopanib. He is starting third-line gemcitabine/docetaxel soon.

## Discussion

The WHO 2021 classification of central nervous system (CNS) tumors classified SFT into three grades [1]. Grade III SFTs, previously known as anaplastic hemangiopericytoma (AHPC), are the most aggressive type, tend to metastasize extracranially, and frequently reoccur [5,6]. The reported metastasis recurrence risk of SFT is 40% during the first five years [7]. The primary metastatic sites are the bone, lung, and liver [7]. At the same time, the recurrence risk of SFTs is estimated to range from 10% to 40% of localized SFTs [7]. Our patient first had an intracranial SFT which was treated successfully and then metastasized to his lungs, right humerus, and second thoracic vertebral body. 

According to Davanzo et al., primary complete surgical resection is the widely accepted treatment strategy for localized oligometastatic disease and the only treatment associated with lower rates of local recurrence [7,8]. Complete surgical resection can achieve a recurrence rate of 8%, however recurrence rate might be higher for patients with longer follow-up duration [8].

When it comes to adjuvant radiotherapy, de Bernardi et al., and other groups have noticed delayed local progression and enhanced local control in patients who had postoperative radiotherapy compared to patients who had not [7,9]. However, these results are based on retrospective analysis, and no prospective studies evaluated the utility of radiotherapy [7,9]. 

Some studies suggested the benefit of chemotherapy in metastatic and advanced SFTs [9]. There are studies that found adding both doxorubicin plus ifosfamide prolongs progression-free survival (PFS) [9]. On the other hand, some studies raised concern regarding the effect of doxorubicin-based regimens on SFTs making these tumors less sensitive to the novel antiangiogenic agents [9]. In addition, gemcitabine-based chemotherapy and paclitaxel-based chemotherapy have been shown to stabilize the disease and prevent its progression in a retrospective study [10]. However, no tumor shrinkage effect was observed after using gemcitabine-based chemotherapy as a first-line for advanced SFTs [10]. Hence, the usefulness of chemotherapy in treating SFT is still questionable. In this case, the patient was treated first by total tumor resection with adjuvant radiotherapy, and after recurrence, he was offered chemotherapy and palliative radiotherapy.

Since SFTs are highly vascularized tumors, novel therapies called antiangiogenics are introduced to block the angiogenesis pathways and hence inhibit tumor proliferation [7]. Several studies used the following angiogenic agents (pazopanib, sunitinib, sorafenib, temozolomide-bevacizumab) for long periods and noticed a mean PFS between 4.7 months to 9.7 months [7]. The first combination drugs introduced were temozolomide and bevacizumab; among the 14 patients who used them 11 had a partial response, two had stable disease and only one had progressive disease [9]. Pazopanib can be used as the first-line in typical SFTs due to its proven efficacy in prospective studies to shorten the PFS, however, it has some cardiotoxic side effects [7,9,11].

Immunotherapy as well is a useful method to target programmed death-ligand 1 protein (PD-L1) which is found to be expressed in all tumors [7]. An anti-PD1 drug called pembrolizumab was used in a patient with pleural SFT for 31 cycles in which he had an almost complete response [7]. Similarly, tumor-infiltrating lymphocytes (TIL) and immune checkpoint biomarkers were found to be expressed in multiple sarcoma types which makes them targets for inhibition by immunotherapy [7]. Overall, the systemic treatment options of SFT is analogue to those of soft-tissue sarcomas due to limited controlled trials that establish a global treatment strategy for SFT [8].

## Conclusions

To date, we believe surgical resection is the best and primary treatment option for SFT. Adjuvant radiotherapy and chemotherapy are debatable; hence further research is needed to evaluate their efficacy. Antiangiogenic therapies, immunotherapies, and IGF-1 Inhibitors are promising drugs that need to be evaluated more in clinical trials. Different modalities of treatment are evolving nowadays, therefore, a multidisciplinary team of pathologists, radiologists, surgical oncologists, radiation oncologists, and medical oncologists are needed to explore new therapeutic models and improve the present therapies to improve disease outcomes. In addition, further clinical trial studies are needed to elucidate the effectiveness of novel drugs in treating SFT.
